# Reviewer acknowledgement

**DOI:** 10.1186/1742-6405-11-8

**Published:** 2014-02-05

**Authors:** Kailash Gupta

**Affiliations:** 1 National Institute of Allergy and Infectious Diseases at the National Institute of Health, Bethesda, Maryland, USA

## Abstract

**Contributing reviewers:**

A peer-reviewed journal would not survive without the generous time and insightful comments of the reviewers whose efforts often go unrecognized. *AIDS Research and Therapy* is very grateful for the support of highly qualified peer reviewers and would like to show its appreciation by thanking the following for their assistance with review of manuscripts for the journal in 2013.

## 

Ramesh Akkina

United States of America

Jintanat Ananworanich

Thailand

Kathryn Anastos

United States of America

Linda Aurpibul

Thailand

Natal Ayiga

South Africa

Melanie Bacon

United States of America

Firouzé Bani Sadr

France

Lealem Bimerew

Ethiopia

Gary Blick

United States of America

Luigi Buonaguro

Italy

Luciana Cardoso

Brazil

Ann Chahroudi

United States of America

Mikhail Chernov

Japan

Ploenchan Chetchotisakd

Timor Leste

Julio Croda

Brazil

Shona Dalal

Uganda

Ruben Dammers

Netherlands

Chris Destache

United States of America

Stephen Dewhurst

United States of America

Joan Duggan

United States of America

Nicolas Durier

Thailand

Andrew Edmonds

United States of America

Stefano Fiorucci

Italy

Luigi Franchi

United States of America

Pierre Frange

France

Joel Gallant

United States of America

Gloria Garrabou

Spain

Davide Gibellini

Italy

Peytavin Gilles

France

Magnus Gisslen

Sweden

Phil Gona

United States of America

Neela Goswami

United States of America

Cecile Goujard

France

Giovanni Guaraldi

Italy

Marguerite Guiguet

France

Anna Dorothee Heemskerk

Vietnam

Natasha Hochberg

United States of America

Michael Horberg

United States of America

Robin Huebner

United States of America

Peter Hunt

United States of America

Jamaiah Ibrahim

Malaysia

Joseph Kagaayi

Uganda

Go Kanzaki

Japan

Arifa Khan

United States of America

Brian Kigozi

Uganda

Anna Kramvis

South Africa

Kenneth Lichtenstein

United States of America

Shuwen Liu

China

Carol Lutz

United States of America

Lut Lynen

Belgium

Frank Maldarelli

United States of America

Wilson Mandala

Malawi

Alexandra Mangili

United States of America

Renato Maserati

Italy

Sheena McCormack

United Kingdom

Kenneth McIntosh

United States of America

John Metcalfe

United States of America

Gerd Mikus

Germany

Jean Michel Molina

France

Patama Monkongdee

Thailand

David Moore

Canada

Giulia Morsica

Italy

Philippe Msellati

Burkina Faso

Eustasius Musenge

South Africa

Celine Nkenfou

Cameroon

Antonio Pacheco

Brazil

William Paxton

Netherlands

Remco Peters

South Africa

Joao Piedade

Portugal

Vinayaka Prasad

United States of America

Wasana Prasitsuebsai

Thailand

Richard Price

United States of America

Jan Prins

Netherlands

Marie Eve Raguenaud

France

Gita Ramjee

South Africa

Peter Rebeiro

United States of America

Steban Ribera

Spain

Elda Righi

Italy

Greg Robbins

United States of America

Lisa Rohan

United States of America

Lisa Ross

United States of America

Stefano Rusconi

Italy

Jesus Salazar Gonzalez

United States of America

Brigitte Sanders

United States of America

Netanya Sandler

United States of America

Rengaswamy Sankaranarayanan

France

Katy Satue`

Spain

Nathaniel Segaren

United Kingdom

Robert Shafer

United States of America

Gerald Sharp

United States of America

Benson Singa

Kenya

Meghan Sise

United States of America

Mark Sonderup

South Africa

Alessandro Soria

Italy

Vincent Soriano

Spain

Cathia Soulie

France

Greg Spear

United States of America

Christoph Spinner

Germany

Marcel Stoeckle

Switzerland

Kazuo Tanaka

Japan

Velislava Terzieva

Bulgaria

Jim Todd

Tanzania

Lydie Trautmann

United States of America

Reinout van Crevel

Netherlands

Koen Van Rompay

United States of America

Stephane Verguet

United States of America

Michael Vinikoor

Zambia

Carole Wallis

South Africa

Jiang Weimin

China

James Whitney

United States of America

Jeffrey Wilusz

United States of America

Lauren Wong

United States of America

Basia Zaba

United Kingdom

Clement Zeh

Kenya

